# Perspectives and Experiences of Patients With Thyroid Cancer at a Global Level: Retrospective Descriptive Study of Twitter Data

**DOI:** 10.2196/48786

**Published:** 2023-08-02

**Authors:** Sununtha Meksawasdichai, Tassanee Lerksuthirat, Boonsong Ongphiphadhanakul, Chutintorn Sriphrapradang

**Affiliations:** 1 Department of Medicine, Faculty of Medicine Ramathibodi Hospital Mahidol University Bangkok Thailand; 2 Research Center, Faculty of Medicine Ramathibodi Hospital Mahidol University Bangkok Thailand

**Keywords:** data mining, internet, natural language processing, sentiment analysis, social media, thyroid neoplasms, twitter, tweet, tweets, neoplasm, neoplasms, cancer, oncology, thyroid, NLP, perspective, perspectives, sentiment, sentiments, experience, experiences

## Abstract

**Background:**

Twitter has become a popular platform for individuals to broadcast their daily experiences and opinions on a wide range of topics and emotions. Tweets from patients with cancer could offer insights into their needs. However, limited research has been conducted using Twitter data to understand the needs of patients with cancer despite the substantial amount of health-related data posted on the platform daily.

**Objective:**

This study aimed to uncover the potential of using Twitter data to understand the perspectives and experiences of patients with thyroid cancer at a global level.

**Methods:**

This retrospective descriptive study collected tweets relevant to thyroid cancer in 2020 using the Twitter scraping tool. Only English-language tweets were included, and data preprocessing was performed to remove irrelevant tweets, duplicates, and retweets. Both tweets and Twitter users were manually classified into various groups based on the content. Each tweet underwent sentiment analysis and was classified as either positive, neutral, or negative.

**Results:**

A total of 13,135 tweets related to thyroid cancer were analyzed. The authors of the tweets included patients with thyroid cancer (3225 tweets, 24.6%), patient’s families and friends (2449 tweets, 18.6%), medical journals and media (1733 tweets, 13.2%), health care professionals (1093 tweets, 8.3%), and medical health organizations (940 tweets, 7.2%), respectively. The most discussed topics related to living with cancer (3650 tweets, 27.8%), treatment (2891 tweets, 22%), diagnosis (1613 tweets, 12.3%), risk factors and prevention (1137 tweets, 8.7%), and research (953 tweets, 7.3%). An average of 36 tweets pertaining to thyroid cancer were posted daily. Notably, the release of a film addressing thyroid cancer and the public disclosure of a news reporter’s personal diagnosis of thyroid cancer resulted in a significant escalation in the volume of tweets. From the sentiment analysis, 53.5% (7025/13,135) of tweets were classified as neutral statements and 32.7% (4299/13,135) of tweets expressed negative emotions. Tweets from patients with thyroid cancer had the highest proportion of negative emotion (1385/3225 tweets, 42.9%), particularly when discussing symptoms.

**Conclusions:**

This study provides new insights on using Twitter data as a valuable data source to understand the experiences of patients with thyroid cancer. Twitter may provide an opportunity to improve patient and physician engagement or apply as a potential research data source.

## Introduction

Social media has become an increasingly influential communication tool, enabling individuals to connect and communicate in real time, regardless of geographic location. Furthermore, social media has emerged as a platform for people to share their thoughts, opinions, and ideas with a global audience. Among the various social media platforms, Twitter stands out, enabling users to share short messages called “tweets” with their followers. These tweets can contain text, images, videos, and links and can be up to 280 characters in length. Users can also interact with tweets by liking, retweeting, or replying to them. Since its launch in 2006, Twitter has become a popular platform for communication, news, and information sharing. Users can follow other users to see their tweets in their Twitter feed and can also use hashtags to categorize their tweets and make them more discoverable to other users. Twitter has also become a powerful tool for businesses, organizations, and public figures to engage with their audiences and promote their brand or message. It is commonly used by journalists, politicians, and celebrities to share their thoughts and opinions on current events. Twitter is available as a website and as a mobile app. According to Twitter’s second-quarter 2022 shareholder letter, the platform had 237.8 million monetizable daily active users worldwide [1].

Twitter plays an important role in public health for several reasons [2,3]. Twitter allows for the rapid spread of information to a large audience. This is particularly important in public health emergencies, such as disease outbreaks or natural disasters, where timely and accurate information can save lives [4,5]. Twitter can be also used for real-time monitoring of disease outbreaks and other public health events [6]. Public health agencies and organizations can use Twitter to disseminate information on health topics, promote healthy behaviors, and engage with the public [7,8]. Some patients and their relatives may choose to share information about their illness on Twitter as a way of raising awareness, connecting with others who are going through similar experiences, or seeking support from the web-based community [9,10]. Analyzing data from Twitter can help identify trends and patterns in public health [11,12]. This can help public health officials better understand public perceptions and attitudes toward specific health issues, which can inform health messaging and interventions.

Many researchers have used the Twitter platform for both enrollment and intervention [2,13,14]. Twitter is indeed unique in its infrastructure and approach to data sharing. One of the key features of Twitter is the ability for any user to follow another user without requiring permission or approval. Additionally, Twitter provides access to almost all of its data. This allows developers and researchers to access and analyze Twitter data for a variety of purposes, such as sentiment analysis, trend analysis, and social network analysis. Twitter not only provides opportunities for sharing experiences between patients and physicians but also the understanding of patients’ perspectives.

Twitter has become a valuable platform for cancer research. Researchers have used Twitter data to study a wide range of topics related to cancer, including public awareness, patient experiences, treatment outcomes, and the use of social media for cancer communication and support. The use of Twitter in cancer research has opened up new possibilities for understanding and addressing the complex challenges associated with cancer and has the potential to improve the lives of patients with cancer and their families. The previous Twitter analysis of patients with cancer found that patients with thyroid cancer had a significantly higher happiness score compared to patients with other types of cancer. This is probably caused by the favorable prognosis and low mortality associated with thyroid cancer, as well as the availability of effective treatment options [10]. Nevertheless, the diagnosis of thyroid cancer frequently triggers intense and immediate emotional responses of shock and fear, evoked by the word “cancer.” Comprehending the experiences of patients upon receiving a diagnosis of thyroid cancer is crucial, as their emotional reactions can significantly influence treatment decision-making and overall quality of life [15].

Researchers have extensively studied breast cancer [16,17], cervical cancer [18,19], lung cancer [20,21], colorectal cancer [22-24], and kidney cancer [25] using Twitter as a valuable data source. However, the number of studies dedicated solely to investigating thyroid cancer through Twitter research is comparatively low in comparison to other cancer types. Typically, when studying thyroid cancer on Twitter, the data are frequently incorporated into broader studies that encompass multiple cancer types [26-28]. In an effort to enhance the data concerning thyroid cancer, we aim to conduct the Twitter analysis using advanced Twitter scraping tool to identify tweets related to thyroid cancer. Through this analysis, we aim to examine the content and sentiments expressed in these tweets on a global scale.

## Methods

### Data Collection and Processing

We searched Twitter for tweets posted between January 1 and December 31, 2020, that included tweets containing the term “thyroid cancer” and collected the data using the Twitter scraping tool Twint. Twint is an advanced Twitter scraping tool written in Python (Python Software Foundation) that allows for scraping tweets from Twitter profiles without using Twitter’s application programming interface. Following the accumulation of raw data, the “pandas” and “contractions” packages were used for data manipulation and cleaning during the preprocessing stage. We collected only tweets in the English language and preprocessed the data by removing irrelevant tweets, duplicates, and retweets. The data were cleaned by removing hyperlinks, URLs, websites, emojis, special characters, numbers, digits, symbols, and any identifiable information. Animal-related tweets were also excluded. In terms of excluding irrelevant tweets, this process was conducted manually, with each tweet being meticulously reviewed by 2 independent reviewers with medical backgrounds (SM and CS). This manual intervention ensured the accuracy and relevance of the data for subsequent analysis. The authors reviewed all tweets and categorized them manually. The categorization process was based on mutual agreement between the 2 reviewers, serving as an internal measure to ensure consistency and reliability.

Twitter users were classified into the groups of patients with thyroid cancer (identified by the presence of personal pronouns such as “I,” “me,” or “my” in their tweets and self-identification as thyroid cancer survivors in their Twitter profiles), patient’s family and friends (whose tweets mentioned their family members or individuals known to have thyroid cancer), medical journals and media (indicated by their Twitter profiles showcasing journal publications or involvement in thyroid cancer media), health personnel (identified through designations such as MD, Dr, doctor, RN, nurse, pharmacist, or PhD in their Twitter profiles), medical health organizations (recognized by their Twitter profiles reflecting hospital names, clinics, or medical institutions), patient community (identified by Twitter profiles associated with thyroid patient networks, groups, or forums), companies (corporation, businesses, or enterprises), and life coaches. Twitter contents were further categorized into distinct groups manually based on key messages, including living with thyroid cancer, treatment (including medication, surgery, and radiotherapy), diagnosis (involving physician consultation, biopsy, fine-needle aspiration, and ultrasound), risk factor and prevention, research (journal publications), entertainment (involving the entertainment industry, actors, movies, TV series), symptoms, knowledge, prevalence and incidence, awareness (related to important days such as Thyroid Awareness Month, National Cancer Day, World Thyroid Day), academic (conferences and meetings), prognosis (regarding the natural history of thyroid disease), and advertisement. Descriptive analytic statistics were used for data analysis.

### Sentiment Analysis

Sentiment analysis is a powerful tool that can be used to understand the emotions toward thyroid cancer [29]. We applied the Transformers package and used the distilbert-base-uncased-finetuned-sst-2-english model for sentiment analysis, which is a natural language processing (NLP) technique designed to analyze emotions based on text data and initially classified the sentiment as positive or negative [30]. This model was trained on a data set of movie reviews, which, similar to tweets, typically involve concise messages. During analysis, the model generates labels indicating the sentiment as either positive or negative, along with an associated confidence score. The tweets that were difficult to classify as positive or negative (confidence score <0.99) were reclassified as neutral. In order to improve the accuracy of our data set, we calibrated the confidence score based on the consensus of 2 reviewers independently reading and interpreting the tweets, and reaching a consensus. The tweets were finally labeled as positive, negative, or neutral regarding thyroid cancer. The percentages for each of the 3 sentiments were calculated.

### Ethics Approval

Although all retrieved tweets are posted publicly on Twitter, our study was approved by the institutional review board (MURA2021/1039). Any personal identifying information was removed to ensure anonymity and protect the identity of the Twitter users.

## Results

**Figure 1:**
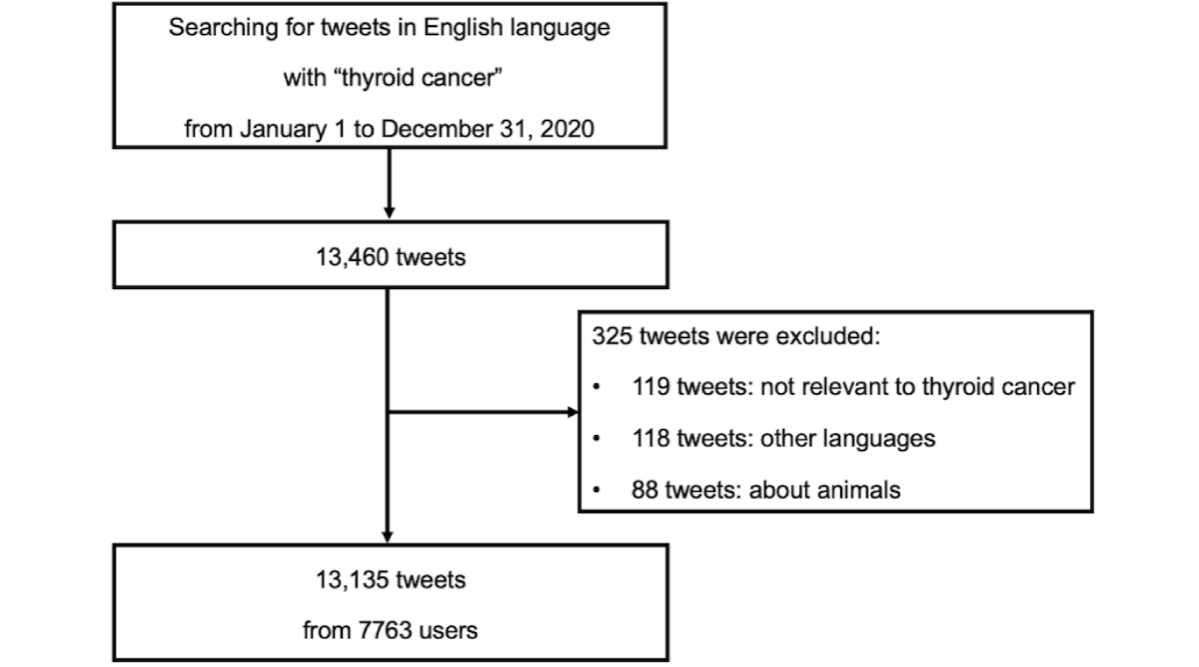
Study flowchart.

Our initial search resulted in a total of 13,460 tweets related to thyroid cancer. We preprocessed the data with the methods previously described. The remaining 13,135 unique tweets from 7763 different users were extracted for analysis (Figure 1).

Of the 13,135 total tweets related to thyroid cancer, the highest percentage of tweets were from patients with thyroid cancer (3225 tweets, 24.6%), followed by patient’s family and friends (2449 tweets, 18.6%), medical journals and media (1733 tweets, 13.2%), health care professionals (1093 tweets, 8.3%), and medical health organizations (940 tweets, 7.2%), respectively. The remaining 24.2% (3176 tweets) could not be identified because of insufficient evidence on their usernames, Twitter profiles, and tweet context (Table 1). Of the 13,135 total tweets related to thyroid cancer, most conversations relevant to thyroid cancer were related to living with cancer (3650 tweets, 27.8%), treatment (2891 tweets, 22%), diagnosis (1613 tweets, 12.3%), risk factors and prevention (1137 tweets, 8.7%), and research (953 tweets, 7.3%). Examples of these tweet contents are shown in Table 2. Focusing on the users and the content relationship, patients as well as families and friends usually talked about living with thyroid cancer, diagnosis, treatment, and symptoms, while health personnel and medical journal and media mainly posted about treatment, diagnosis, research, and knowledge (Table 3).

**Table 1:** Definitions of Twitter users and frequency of tweets related to thyroid cancer.

Twitter users	Definitions	Tweets, n (%)
Patients	Thyroid cancer patients (the tweets usually have “I,” “me,” or “my” in the phrase)	3225 (24.6)
Patient’s family and friends	Family members, relatives, friends, and colleagues	2449 (18.6)
Medical journals and media	Medical publications and press	1733 (13.2)
Health care professionals	Physician, nurse, and allied health professionals	1093 (8.3)
Medical health organizations	Hospital, clinic, and medical institution	940 (7.2)
Patient community	Patient network, group, society, and forum	352 (2.7)
Company	Corporation, business, and enterprise	137 (1)
Life coach	Personal coach, wellness coach, and success coach	30 (0)
Inconclusive	Indistinguishable to determine the type of user	3176 (24.2)

**Table 2:** Twitter contents related to thyroid cancer.

Twitter contents	Examples	Tweets, n (%)
Living with thyroid cancer	I found a reddit community for thyroid cancer and I’m talking about my rare type on it and giving my WHOLE story like as detailed as I can. It’s like I’m writing a novel lol. Don't ever tell a person with thyroid cancer that they have the best kind of cancer. I'm in pain almost every day.	3650 (27.8)
Treatment	Feels like I’ve been training for social distancing for years. I’ve had isolation during treatments of radioactive iodine (thyroid cancer), have physical limitations that make it difficult to get around. So, here’s my advice to the newbies: #COVID2019 Today marks my 3rd year post operation from my thyroid cancer. Will share some of my before and after photos in my stories. I’m just so happy that I’m alive and healthy from my own perspective.	2891 (22)
Diagnosis	My throat can hurt and the first thing on Google is throat cancer, thyroid cancer, or swollen lymph nodes from lymphoma or HIV Real pissed my old endocrinologist never did an ultrasound on my thyroid or even TOLD me that Hashimoto’s makes me way more susceptible to thyroid cancer. My new doc was confused why I’d never had a thyroid ultrasound for that exact reason.	1613 (12.3)
Risk factor and prevention	Listening to the father-in-law decided to stop his diabetic medication because it may cause thyroid cancer. Thyroid cancer diagnoses are up to three times more common in 9/11 first responders than the general population. However, the increased cancer rate may be due to over screening, according to a new study in @JAMAInternalMed.	1137 (8.7)
Research	Lilly Opens Phase 3 Clinical Trial for Selpercatinib (LOXO-292) in RET-Mutant Medullary Thyroid Cancer Fukushima Nuclear Disaster | Increased Thyroid Cancer in U.S.	953 (7.3)
Entertainment	I bet this is when Ezekiel tells Carol about his thyroid cancer #TheWalkingDead Completed watching 'Dil Bechara', Kizie Basu is fighting thyroid cancer when she meets Immanuel Rajkumar Junior or Manny, who has previously suffered from osteosarcoma and is in remission.	582 (4.4)
Symptoms	I struggle to lose weight. I have an under active thyroid due to thyroid cancer. Some people can't help being overweight. I've gained a lot of weight because I had thyroid cancer and it caused me to gain almost 75 lbs. I'm a big lady. 250 lbs. My husband says it's just more to love that's all. But I can't bear to look at myself in the mirror.	549 (4.2)
Knowledge	The Different Types of Thyroid Cancer Medscape: Review this updated thyroid cancer reference.	531 (4)
Prevalence and incidence	The number of people diagnosed with #thyroid cancer has more than doubled worldwide since 1990. Much of the increase has been fueled by a rapid rise of #cancer cases in countries in South-East Asia, which accounted for more than 40% of global diagnoses. Thyroid cancer remains the highest prevailing endocrine malignancy, and its incidence rate has progressively increased in the previous years. #Thyroidcancer	355 (2.7)
Awareness	Thyroid Awareness Month calls attention to thyroid conditions such as hypothyroidism, hyperthyroidism, Graves’ disease, Hashimoto’ disease, goiter, thyroid nodules, and thyroid cancer. Thyroid Cancer Awareness Month ends today but the fight against #thyroidcancer is far from over. Help us raise awareness and continue the conversation. #CheckYourNeck #ThyroidCancerAwareness	339 (2.6)
Academic	Join us to clarify coding misconceptions when collecting cancer data in your role as a cancer registrar. We will be discussing the nuances of abstracting THYROID CANCER. You won't want to miss! #Cancerregistry #Data #Healthcare Fukushima Thyroid Cancer Symposium live stream 3 February 2020	219 (1.7)
Prognosis	Papillary and Follicular thyroid is in general excellent. Factors include age, aggressiveness, metastasis. Medullary Thyroid cancer may have a good prognosis too but should be evaluated for other endocrine problems. Anaplastic thyroid cancer may have a poor outcome. Thyroid cancer has a 99% cure rate. Learn to spot the signs and what to do next if you notice something unusual in your throat or neck: #cancer #cancercare #earlydetection #oncology #Detroit	214 (1.6)
Advertisement	If you live within 50km of #Pickering nuclear you can order your free KI pills here to help protect against thyroid cancer Thyroid Cancer: A Guide for Patients 3rd Edition Now Available. For details and ordering information, visit our website: #ThyroidCancer #ThyCa #ThyCa4Life	102 (0.8)

**Table 3:** Tweet contents related to thyroid cancer classified by various types of Twitter users. The percentage represents the row percentage.

Twitter users	Living with thyroid cancer, n (%)	Treatment, n (%)	Diagnosis, n (%)	Risk factors and prevention, n (%)	Research, n (%)	Entertainment, n (%)	Symptoms, n (%)	Knowledge, n (%)	Prevalence and incidence, n (%)	Awareness, n (%)	Academic, n (%)	Prognosis, n (%)	Advertisement, n (%)
Patients	1501 (47)	729 (23)	503 (16)	72 (2)	11 (0)	79 (2)	251 (8)	8 (0)	4 (0)	41 (1)	2 (0)	19 (1)	5 (0)
Inconclusive	278 (9)	494 (16)	395 (12)	603 (19)	269 (9)	404 (13)	79 (3)	171 (5)	161 (5)	128 (4)	52 (2)	98 (3)	44 (1)
Family and friends	1423 (58)	483 (20)	295 (12)	50 (2)	1 (0)	53 (2)	120 (5)	0 (0)	3 (0)	3 (0)	1 (0)	15 (1)	1 (0)
Medical journals and media	43 (3)	502 (29)	174 (10)	150 (9)	435 (25)	31 (2)	37 (2)	154 (9)	80 (5)	37 (2)	47 (3)	37 (2)	6 (0)
Health care professionals	196 (18)	280 (26)	145 (13)	88 (8)	121 (11)	8 (1)	17 (2)	104 (10)	37 (3)	22 (2)	31 (3)	29 (3)	14 (1)
Medical health organization	72 (8)	294 (31)	64 (7)	151 (16)	81 (9)	3 (0)	32 (3)	62 (7)	57 (6)	53 (6)	48 (5)	15 (2)	8 (1)
Patient community	126 (36)	58 (17)	23 (7)	5 (1)	14 (4)	3 (1)	10 (3)	16 (5)	10 (3)	45 (13)	35 (10)	1 (0)	6 (2)
Company	7 (5)	44 (32)	13 (10)	18 (13)	20 (15)	1 (1)	2 (2)	8 (6)	3 (2)	9 (7)	3 (2)	0 (0)	9 (7)
Life coach	4 (13)	7 (23)	1 (3)	0 (0)	0 (0)	0 (0)	1 (3)	7 (23)	0 (0)	1 (3)	0 (0)	0 (0)	9 (30)

**Figure 2:**
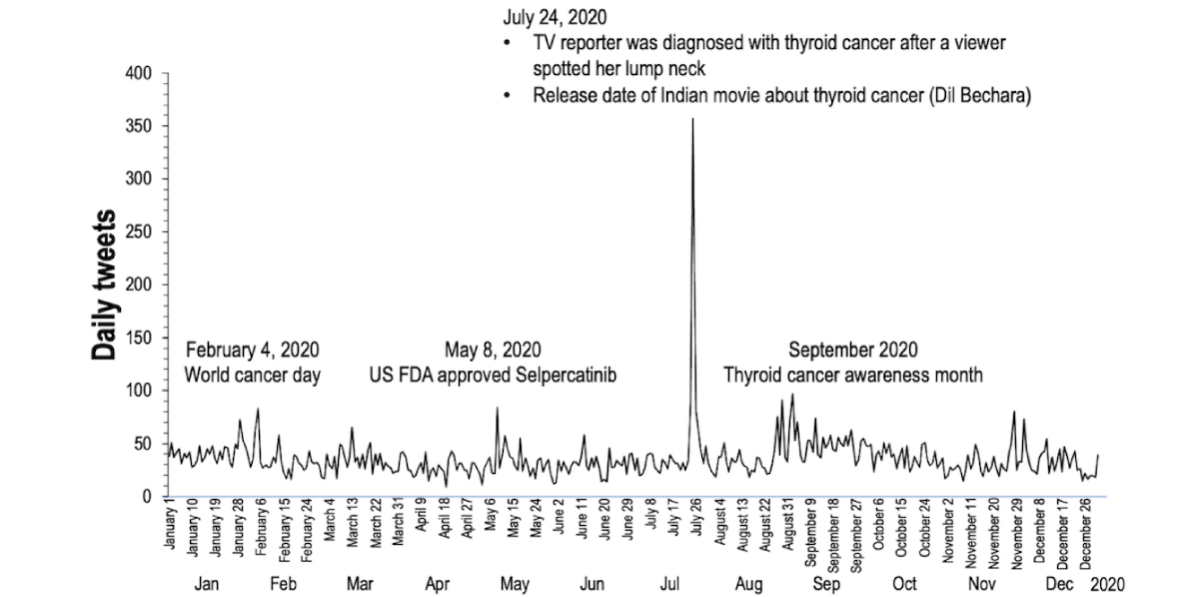
Tweets over the year 2020. US FDA: US Food and Drug Administration.

In 2020, an average of 36 thyroid cancer–related tweets were posted each day (Figure 2). The number of tweets posted on July 24, 2020 (357 tweets), was 10 times higher than the average because the United States TV reporter announced that she was diagnosed with thyroid cancer after a viewer spotted a lump on her neck. On the same day, “Dil Bechara,” an Indian movie adapted from “The Fault in Our Stars” the female lead suffered from thyroid cancer, was released on the streaming service. Throughout the year, several other days recorded higher tweet volumes of 80-100 tweets per day. Examples include February 4, 2020 (World Cancer Day), May 8, 2020 (United States Food and Drug Administration approval of Selpercatinib for advanced RET-driven lung and thyroid cancers), and September 1, 2020 (the first day of Thyroid Cancer Awareness Month).

Of the 13,135 total tweets related to thyroid cancer, the sentiment analysis revealed that 7025 (53.5%) tweets were categorized as neutral statements, while 4299 (32.7%) tweets were labeled as negative emotions. Tweets from patients with thyroid cancer had the highest proportion of negative emotion, with 1385 out of 3225 (42.9%) tweets, as shown in Table 4. Notably, tweets discussing symptoms showed the highest prevalence of negative emotions (Table 5). In the year 2020, the COVID-19 outbreak began. There were 427 tweets that relate to thyroid cancer and COVID-19. The highest tweet rate was in March 2020, according to the declaration of COVID-19 as a global pandemic by World Health Organization on March 11, 2020. The predominant topic among COVID-19–related tweets was living with thyroid cancer, accounting for 226 (52.9%) tweets. From sentiment analysis, negative emotions were found in almost half (210/427 tweets, 49.2%) of COVID-19–related tweets compared with 32.2% (4089/12,708) of tweets in non–COVID-19–relevant tweets.

**Table 4:** Sentiment analysis of tweets related to thyroid cancer according to Twitter users.

Twitter users	Positive, n (%)	Neutral, n (%)	Negative, n (%)
Patients	583 (18.1)	1257 (39)	1385 (42.9%)
Family and friends	525 (21.4)	991 (40.5)	933 (38.1)
Medical journals and media	155 (8.9)	1285 (74.1)	293 (16.9)
Health care professionals	119 (10.9)	737 (67.4)	237 (21.7)
Medical health organization	103 (11.)	596 (63.4)	241 (25.6)
Patient community	84 (24)	197 (56)	71 (20)
Company	11 (8)	108 (78.8)	18 (13)
Life coach	8 (27)	18 (60%)	4 (13)
Inconclusive	223 (7)	1836 (57.8)	1117 (35.2)

**Table 5:** Sentiment analysis of tweet contents related to thyroid cancer.

Tweet contents	Positive, n (%)	Neutral, n (%)	Negative, n (%)
Living with thyroid cancer	835 (22.9)	1642 (45)	1173 (32.1)
Treatment	376 (13)	1570 (54.3)	945 (32.7)
Diagnosis	160 (9.9)	807 (50)	646 (40)
Risk factors and prevention	33 (3)	635 (55.8)	469 (41.2)
Research	92 (10)	724 (76)	137 (14.4)
Entertainment	66 (11)	273 (46.9)	243 (41.8)
Symptoms	36 (7)	209 (38.1)	304 (55.4)
Knowledge	37 (7)	342 (64.4)	152 (28.6)
Prevalence and incidence	19 (5)	249 (70.1)	87 (24)
Awareness	56 (16)	243 (71.7)	40 (12)
Academic	52 (24)	147 (67.1)	20 (9)
Prognosis	25 (12)	124 (57.9)	65 (30)
Advertisement	24 (23)	60 (59)	18 (18)

## Discussion

### Principal Findings

In this study, we explored Twitter users and topics of tweet content associated with thyroid cancer in the year 2020. The majority of tweets (5674/13,135, 43.2%) were contributed by individuals who identified as patients or were related to them as family members and friends. A combined contribution of tweets (3766/13,135, 28.7%) was observed from sources such as medical journals and media, health care professionals, and medical health organizations.

The most popular topics of tweet contents pertained to coping with thyroid cancer and its management. According to the sentiment analysis, tweets related to thyroid cancer exhibited a greater prevalence of negative emotions, particularly among individuals diagnosed with thyroid cancer.

### Comparison With Prior Work

In comparison to 2014 data [10], there was a significant increase in the proportion of tweets authored by patients, rising from 11.8% to 24.6% of the total tweets. This indicates a growing interest in patient-centered discussions about thyroid cancer on Twitter. However, it is important to acknowledge that the previous study only included geotagged tweets from the United States, which may not provide a representative view at the global level [10]. Nonetheless, this highlights the potential of social media as a valuable tool for thyroid cancer, enabling them to connect, find support, access information, and raise awareness. When used responsibly, social media can substantially enhance the patient experience and contribute to improved outcomes throughout their cancer journey.

Our findings demonstrate the potential of Twitter as a robust medium for individuals with thyroid cancer to share personal experiences, ranging from diagnosis to therapeutic interventions. Through sharing their experience, patients with thyroid cancer can raise awareness about the difficulties associated with cancer and motivate others who may be confronting similar challenges [31,32]. Moreover, health care providers, media outlets, and health care organizations play a pivotal role in disseminating information, articles, and research findings [16,19,33]. By following relevant organizations and individuals on Twitter, patients with thyroid cancer can stay updated on the latest advances in cancer treatment and research, as well as discover resources such as support groups, financial assistance programs, and clinical trials [34-36]. Our results at a worldwide level were consistent with the earlier data reported in the United States [37].

Twitter can also serve as a platform for expressing negative affective states, such as fear, anxiety, anger, frustration, sadness, grief, isolation, and loneliness, which are commonly experienced in the context of cancer diagnosis and treatment. While a previous study found that patients with thyroid cancer had a high average word happiness value [10]; however, this study found that almost half of tweets from patients with thyroid cancer displayed negative sentiments. The negative issues identified in this study were mainly related to symptoms experienced by patients, which caused significant distress. Another significant concern was related to the risk factors associated with thyroid cancer, with many patients expressing uncertainty about the causes of their condition, including the potential impact of nuclear disasters and events like the September 11 attacks. Patients and their families were also greatly impacted by the diagnosis of thyroid cancer, with many expressing shock and disbelief upon receiving the news. While some patients in the prior study reported feeling comfortable with their diagnosis, others felt confused and ignored, particularly in cases where they were told that thyroid cancer was a “good cancer” by multiple sources [38-40]. Based on the previous study conducted on breast cancer, the decline in negative attitudes toward cancer observed across various social media platforms, including Twitter [17], could be indicative of improved efforts by health organizations and agencies to educate the public on cancer, including its prevention, treatment, and management. Social media could serve as a valuable source of information to gain insight into the layperson’s perceptions and attitudes regarding topics related to thyroid cancer. For instance, an examination of tweets has highlighted postoperative weight gain as a significant concern. However, existing evidence indicates that any weight gain among these patients might be linked to the natural process of aging rather than the surgical intervention itself [41]. This knowledge can facilitate informed conversations between health care providers and patients, enabling them to set realistic postoperative expectations and address misconceptions about weight gain after thyroid surgery.

The launch of the movie about thyroid cancer and the news reporter’s announcement of her thyroid cancer diagnosis triggered a surge in the number of tweets. Consistent with previous studies, social media influencers played a significant role in public conversations [42,43]. This study demonstrated the notable involvement of celebrities, public figures, and health care personnel in disseminating health messages through social media.

Our findings support the analysis of Twitter data for implications of public health, clinical practice, and future research. Analyzing Twitter data can provide valuable insights into public perceptions, attitudes, and concerns about health topics, including thyroid cancer. This information aids public health officials in understanding population needs. It can inform the development of targeted health promotion campaigns, interventions, and educational materials to address specific concerns raised by the public on Twitter. In addition, monitoring trends and discussions on Twitter can help identify emerging health issues and facilitate timely public health responses. For clinical practice, studying Twitter data deepens health care providers’ understanding of patient experiences, treatment preferences, and impact on quality of life, enabling more patient-centered care and tailored support resources. Twitter studies also serve as a valuable data source for exploring research questions, uncovered patterns, and generating hypotheses. By examining large volumes of real-time user-generated content, researchers can uncover new patterns, trends, and associations. These data can be used to generate hypotheses, inform study design, and guide the development of research interventions. In addition, the use of social media data can complement traditional research methods, providing a more comprehensive understanding of health issues and allowing for a broader reach and engagement with diverse populations.

### Strengths and Limitations of This Study

The main strength of this research was the year-long study period, which reduced the potential for time period bias and allowed for the analysis of different types of tweets at various times. However, there were several limitations to this study, including imperfect data collection. For instance, only public tweets were accessible, and private Twitter accounts were not included. In addition, the use of only a single search term “thyroid cancer” may have resulted in selection bias. We recognize the limitation associated with the absence of geographic data. The origin of the tweets could potentially lead to variations in the data across different regions or countries. Demographic data were not gathered, and some tweets were unclear. It is also important to note that Twitter users tend to be younger, which may not accurately represent the broader population of patients with thyroid cancer. It is essential to acknowledge that while NLP is an exceptionally powerful tool, it also has inherent limitations. For instance, NLP primarily operates on textual data and may encounter difficulties in accurately interpreting ambiguous phrases, slang, and sarcasm. These factors can occasionally result in inaccuracies in sentiment classification. The categorization of tweets was conducted through a meticulous manual assessment process, devoid of a formal codebook. Regrettably, no interrater reliability was evaluated. Nevertheless, the categorization was determined based on mutual agreement between the 2 reviewers, both of whom have medical backgrounds. Furthermore, since the data were collected during the COVID-19 pandemic, negative emotions expressed in the tweets may have been influenced by disruptions in cancer care and the risk of COVID-19 infection and complications [44,45].

### Conclusions

Twitter is a valuable social media platform for health research due to the wealth of data available that offers insights into users’ perspectives. This study provides essential information to understand the thoughts and emotions of patients with thyroid cancer, which can be helpful in the development of medical services and better patient care. Additionally, this study highlights the potential of Twitter as a platform for health care providers and organizations to disseminate health information and communicate with patients. Collaborating with public figures and social media influencers can enhance the reach and effectiveness of health campaigns and messaging.

## Abbreviations

NLP: natural language processing

## Data Availability

The data sets generated and analyzed during this study are available from the corresponding author upon request.
